# HECIL: A Hybrid Error Correction Algorithm for Long Reads with Iterative Learning

**DOI:** 10.1038/s41598-018-28364-3

**Published:** 2018-07-02

**Authors:** Olivia Choudhury, Ankush Chakrabarty, Scott J. Emrich

**Affiliations:** 1 Postdoctoral Researcher, IBM Research, Cambridge, MA 02142 USA; 2 Visiting Research Scientist, Mitsubishi Electric Research Laboratories, Cambridge, MA 02139 USA; 3 Associate Professor, Department of Electrical Engineering and Computer Science, University of Tennessee, Knoxville, TN 37996 USA

## Abstract

Second-generation DNA sequencing techniques generate short reads that can result in fragmented genome assemblies. Third-generation sequencing platforms mitigate this limitation by producing longer reads that span across complex and repetitive regions. However, the usefulness of such long reads is limited because of high sequencing error rates. To exploit the full potential of these longer reads, it is imperative to correct the underlying errors. We propose HECIL—Hybrid Error Correction with Iterative Learning—a hybrid error correction framework that determines a correction policy for erroneous long reads, based on optimal combinations of decision weights obtained from short read alignments. We demonstrate that HECIL outperforms state-of-the-art error correction algorithms for an overwhelming majority of evaluation metrics on diverse, real-world data sets including E. coli, S. cerevisiae, and the malaria vector mosquito A. funestus. Additionally, we provide an optional avenue of improving the performance of HECIL’s core algorithm by introducing an iterative learning paradigm that enhances the correction policy at each iteration by incorporating knowledge gathered from previous iterations via data-driven confidence metrics assigned to prior corrections.

## Introduction

Current advances in next-generation sequencing (NGS) have fueled genomics-driven research by inexpensively generating highly accurate ‘reads’ or DNA sequence fragments. Second-generation sequencing technologies, for example Illumina^1^ and 454 pyro-sequencing^2^, generate short reads that are sometimes not ideal for downstream applications such as assembling complex genomes^3^. To ameliorate this issue, third-generation sequencing techniques introduced by Pacific Biosciences^4,5^ and Oxford Nanopore^6,7^ generate significantly longer reads. These long reads typically contain thousands of base-pairs^8^ and are not subject to amplification or compositional biases often exhibited by second-generation sequencing^9^. Long reads also overcome issues associated with repetitive regions and large transcript isoforms. In spite of these significant advantages, a critical limitation of long reads produced by third-generation sequencing methods is that they generally exhibit high error rates: for example, up to 20% error has been reported using PacBio^10,11^, and up to 35% error using Oxford Nanopore^12^.

Various correction algorithms have been proposed for reducing the currently high error rates prevalent in long reads. For example, HGAP^13^ is a self-correcting algorithm (that is, it does not rely on additional sequencing data) that performs correction by computing multiple alignments of high coverage long reads. Another class of correction algorithms rely on short reads generated from the same (or related) samples, and is therefore referred as *hybrid correction algorithms*. An example of such a hybrid correction algorithm is the Nanocorr algorithm^12^ in which high-quality Illumina MiSeq reads are used to correct Oxford Nanopore reads. Popular hybrid correction algorithms for PacBio data include: LSC^3^, PacBioToCA^8^, LoRDEC^14^, proovread^15^, and CoLoRMap^16^. Most of the methods listed here do not systematically utilize localized information such as base quality of the short reads or variant information between individuals. The importance of incorporating base quality in correcting noisy sequence data is well-known^17^, and serves as a primary motivation for the present work.

Herein, we propose a hybrid error correction framework that we refer to as HECIL. The proposed algorithm comprises two components:

1. a *core algorithm* that selects a correction policy by leveraging an optimal combination of decision weights based on base quality and mapping identity of aligned short reads; and,
2. an *iterative procedure* that enables learning from data generated in previous iterations to improve subsequent alignment and corrections.

We compare HECIL’s core algorithm to existing hybrid correction algorithms on real prokaryotic and eukaryotic data and, for an overwhelming majority of evaluation metrics (related to both alignment and assembly), show that HECIL’s core algorithm outperforms its competitors. The iterative procedure further improves the quality of error correction both in terms of alignment and assembly-based metrics by incorporating knowledge derived from high-confidence corrections made in prior iterations. We speculate that the proposed iterative learning formalism can be incorporated into other contemporary hybrid error correction algorithms to improve performance, at the expense of total execution time.

## Results

All experiments in this section were run on Dell PowerEdge R815 servers with AMD Opteron processor 6378, Quad 16 core 2.4 GHz CPU, 32 cores, and 512 GB RAMs. We use the Unix time command to record the runtime and memory usage of each tool. We test the performance of HECIL on real datasets of varying size: the bacterial genome of *Escherichia coli*, the fungal genome of *Saccharomyces cerevisiae*, and the malaria vector genome of *Anopheles funestus*. We explore benchmark data of PacBio-sequenced long reads, Illumina-sequenced short reads, and reference genomes of *E. coli* and *S. cerevisiae*, as used by the state-of-the-art correction tool CoLoRMap^16^. We filter long reads of *E. coli* to exclude reads shorter than 100 bp, creating a final set of 33,360. The corresponding short reads comprise 22,720,100 sequences. We use the strain K-12 substr. MG1655 for our alignment-based validation of HECIL. To test *S. cerevisiae* data, we use 1,758,169 long reads with 4,503,422 short reads. The reference genome of strain S288C is 12.2 Mbp in size. We obtain long reads for *A. funestus*, comprising data from 44 flowcells, ranging between 59,937 and 244,754 reads. Due to the high computational effort required by proovread and CoLoRMap to correct the reads of all flowcells, we present a comparative analysis based on a representative (albeit arbitrary) selection of three flowcells: 1, 4, and 16. Short read sequences consists of 37,797,235 reads. The reference genome of strain Fumoz (GenBank assembly accession: GCA_000349085.1) is used for validating corrections. Finally, we test HECIL on the long reads of *E. coli* generated by the newest Single Molecule, Real-Time sequencer, the Sequel System^18^.

### Evaluation metrics

#### *k*-mer-based

We employ the widely-used *k*-mer counting tool Jellyfish^19^ to compute the number of unique *k*-mers obtained after each correction algorithm. Since errors in long reads are uniformly distributed across their length, large numbers of uncorrected errors often greatly inflate the number of unique *k*-mers observed. Further, it is known^20^ that the set of common *k*-mers between the highly accurate short reads and the erroneous long reads are crucial in improving the quality of data for downstream analysis. Therefore, a correction algorithm that reduces the number of unique *k*-mers while increasing the number of valid *k*-mers is desirable. Supplementary Figure S1 gives an illustrative example of this idea based on *A. funestus*.

#### Alignment-based

After each method of correction, we align corrected long reads to its reference genome using BLASR^21^. In addition to computing the number of aligned reads and aligned bases, we evaluate matched bases, that is, the ratio of total number of matched bases and length of sequences in the long reads. We calculate percent identity (PI) by the ratio of matches to alignment length.

#### Assembly-based

One of the most important downstream applications of long reads is *de novo* genome assembly. For this purpose, we use the assembler Canu^22^, specifically designed for noisy long reads. We then use QUAST^23^ to evaluate assembly quality. We measure total number of contigs, length of the longest contig, and total length (total number of bases in the assembly). We report the values of N50 (minimum length such that contigs of that length or longer consists half the assembly), and NG50 (minimum length such that contigs of that length or longer consists half the reference assembly). As recommended in prior art^22^, we further measure accuracy by aligning the assembled genome to the reference genome using MUMMer’s dnadiff tool^24^. In this context, we compute percent of aligned bases (with respect to reference and query) and average identity of 1-to-1 alignment blocks (with respect to reference and query).

### Comparative analysis

We compare the performance of HECIL with cutting-edge hybrid error correction tools such as proovread-2.14.0, LoRDEC-0.6, and CoLoRMap. We use the above-mentioned *k*-mer-based, alignment-based, and assembly-based metrics to assess the performance of each approach. The comparative results for *k*-mer-based and alignment-based parameters are presented in Table 1. We report the parameters before correction (original) and after each method of error correction.

**Table 1:** Comparison of *k*-mer-based and alignment-based metrics (with % improvement) evaluated from testing *E. coli*, *E. coli* (Sequel-sequenced), *S. cerevisiae*, and *A. funestus* on proovread, LoRDEC, CoLoRMap, and HECIL.

Data	Evaluation Metric	Original	proovread	LoRDEC	CoLoRMap	HECIL (Iter 1)	HECIL (Iter 5)
*E. coli*	# unique *k*-mers	81,523,648	78,925,288 (3.1)	80,708,419 (1.0)	80,399,425 (1.3)	**78,693,704 (3.4)**	**77,617,181 (4.7)**
	# valid *k*-mers	14,531,881	11,463,127 (−21.1)	10,240,970 (−29.5)	15,026,950 (3.4)	**15,973,826 (9.9)**	**16,413,012 (12.9)**
	# aligned reads	31,071	23,453 (−24.5)	30,837 (−0.7)	31,271 (0.6)	**31,332 (0.8)**	**31,401 (1.0)**
	# aligned bases	86,642,500	71,320,858 (−17.6)	79,365,407 (−8.4)	83,344,272 (−3.8)	**87,582,014 (1.0)**	**88,809,361 (2.5)**
	% matched bases	76.9	87.9 (14.3)	85.2 (10.7)	87.5 (13.7)	**88.4 (14.9)**	**89.4 (16.2)**
	PI	94.8	**99.7 (5.1**)	99.4 (4.8)	99.2 (4.6)	**99.7 (5.1)**	**99.8 (5.27)**
*E. coli* (Sequel)	# unique *k*-mers	1,982,480,568	84,739,287 (95.7)	86,825,382 (95.6)	85,031,655 (95.7)	**82,017,841 (95.8)**	**80,925,018 (95.9)**
	# valid *k*-mers	11,890,472	11,365,013 (−4.4)	10,167,397 (−14.4)	12,626,801 (6.1)	**16,957,253 (95.8)**	**17,826,131 (95.9)**
	# aligned reads	1,158,421	910,384 (−21.4)	1,161,432 (0.2)	1,189,253 (2.6)	**1,201,357 (3.7)**	**1,382,839 (19.3)**
	# aligned bases	4,343,460,105	3,963,123,749 (8.7)	4,471,081,390 (2.9)	4,416,369,371 (1.6)	**4,698,531,714 (8.1)**	**4,927,137,639 (13.4)**
	% matched bases	85.1	93.1 (9.4)	92.8 (9.0)	93.7 (10.1)	**95.6 (12.3)**	**97.1 (14.1)**
	PI	85.0	93.1 (9.5)	92.8 (9.1)	93.7 (10.2)	**95.6 (12.4)**	**97.2 (14.3)**
*S. cerevisiae*	# unique *k*-mers	1,870,396,869	1,871,451,237 (−0.0)	1,868,238,946 (0.1)	1,869,232,456 (0.0)	**1,867,828,519 (0.1)**	**1,865,148,289 (0.2)**
	# valid *k*-mers	36,904,129	32,436,294 (−12.1)	30,534,546 (−17.2)	37,797,300 (2.4)	**39,452,743 (6.9)**	**40,971,328 (11.0)**
	# aligned reads	224,694	222,976 (−0.7)	221,692 (−1.3)	223,641 (−0.4)	**346,242 (54.0)**	**346,307 (54.1)**
	# aligned bases	1,229,724,663	1,205,706,114 (−1.9)	1,171,490,123 (−4.7)	1,207,729,568 (−1.7)	**1,247,616,674 (1.4)**	**1,249,303,521 (1.5)**
	% matched bases	78.8	83.1 (5.4)	83.4 (5.8)	85.6 (8.6)	**85.6 (8.6)**	**86.5 (9.7)**
	PI	93.8	96.3 (2.6)	98.3 (4.8)	98.3 (4.8)	**98.6 (5.1)**	**98.9 (5.4)**
*A. funestus # 1*	# unique *k*-mers	692,831,731	649,989,172 (6.1)	653,931,808 (5.6)	662,366,838 (4.4)	**649,764,906 (6.2)**	**648,091,381 (6.4)**
	# valid *k*-mers	211,908,809	172,074,427 (−18.8)	229,625,736 (8.3)	222,195,325 (4.8)	**242,957,349 (14.6)**	**244,317,225 (15.2)**
	# aligned reads	190,217	94,536 (−50.3)	**190,240 (0.0)**	190,166 (−0.0)	190,229 (0.0)	**191,245 (0.5)**
	# aligned bases	671,881,278	401,850,047 (−40.1)	655,072,426 (−2.5)	660,848,583 (−1.6)	**676,055,060 (0.6)**	**678,092,137 (0.9)**
	% matched bases	84.0	81.4 (−3.1)	83.1 (−1.0)	82.1 (−2.2)	**85.1 (1.3)**	**87.9 (4.6)**
	PI	94.5	96.8 (2.4)	95.6 (1.1)	97.1 (2.7)	**97.8 (3.4)**	**98.5 (4.2)**
*A. funestus # 4*	# unique *k*-mers	216,327,700	**205,053,236 (5.2)**	205,883,182 (4.8)	206,986,374 (4.3)	205,064,188 (5.2)	**203,997,977 (5.7)**
	# valid *k*-mers	80,612,612	72,716,589 (−9.8)	82,568,831 (2.4)	81,027,437 (0.5)	**83,788,157 (3.9)**	**84,529,123 (4.8)**
	# aligned reads	59,163	32,726 (−44.6)	59,165 (0.0)	59,159 (−0.0)	**59,177 (0.0)**	**59,306 (0.24)**
	# aligned bases	231,326,514	149,049,154 (−35.5)	234,098,182 (1.2)	233,435,402 (0.9)	**235,620,667 (1.8)**	**237,428,249 (2.6)**
	% matched bases	86.3	83.2 (−3.5)	87.0 (0.8)	85.6 (−0.8)	**87.2 (1.0)**	**89.3 (3.48)**
	PI	94.3	96.9 (2.7)	96.6 (2.4)	97.2 (3.0)	**97.7 (3.6)**	**98.4 (4.3)**
*A. funestus # 16*	# unique *k*-mers	265,998,542	250,267,133 (5.9)	252,291,701 (5.1)	254,293,778 (4.4)	**249,528,780 (6.1)**	**248,471,673 (6.5)**
	# valid *k*-mers	96,317,177	86,396,798 (−10.3)	106,713,483 (10.7)	101,431,900 (5.3)	**109,954,860 (14.6)**	**110,798,014 (15.0)**
	# aligned reads	73,779	43,530 (−41.0)	73,757 (−0.0)	73,750 (−0.0)	**73,790 (0.0)**	**74,111 (0.45)**
	# aligned bases	278,976,792	190,054,632 (−31.8)	280,699,552 (0.6)	280,831,201 (0.6)	**282,244,589 (1.1)**	**283,981,841 (1.7)**
	% matched bases	84.3	82.7 (−1.9)	85.6 (1.5)	84.5 (0.2)	**86.1 (2.1)**	**87.5 (3.8)**
	PI	94.8	96.9 (2.2)	96.3 (1.5)	97.4 (2.7)	**98.0 (3.3)**	**98.6 (4.0)**

For the case of HECIL, metrics are reported before and after using the iterative learning algorithm; specifically, iteration 1 (the core algorithm) and iteration 5 (with four rounds of learning) are shown.

As expected, CoLoRMap^16^ performs better than proovread and LoRDEC when tested on *E. coli* (PacBio and Sequel-sequenced) and *S. cerevisiae*. However, long reads corrected by the core algorithm of HECIL (iteration 1) generate the lowest number of *k*-mers for each of these datasets (with the exception of the data set *A. funestus* - flowcell 4, although it is still comparable to the best results obtained using proovread), outperforming CoLoRMap. For all data sets, HECIL consistently produces more valid *k*-mers: since an increase in valid *k*-mers indicates higher consensus to the accurate short reads, producing more valid *k*-mers implies that HECIL generates corrected long reads with higher accuracy than its competitors. HECIL also produces the highest number of aligned bases, reads, and highest percent identity.

We also study the effect of HECIL on assembly-based metrics; the results are tabulated in Table 2. HECIL’s core algorithm (Iter 1) generates more contiguous assembled long reads compared to the existing tools, except for *E. coli* and yeast where the performance is identical to CoLoRMap. When other metrics such as the size of the longest contig and the number of bases in the assembled data are compared, we exhibit the best performance unequivocally. Standard assembly quality parameters like N50 and NG50 have highest values after using HECIL for correction, and the assembled genomes of HECIL have higher aligned bases and 1-to-1 alignment identity. Note that the proportion of aligned bases in the reference genome with respect to the query genome is low because we use a subset of mosquito flowcell data. For highly heterozygous samples such as insects like mosquitoes^25^, low frequency bases in aligned short reads may indicate inherent variation that are not necessarily sequencing errors. Correction algorithms that solely rely on a consensus call or majority vote often discard these heterogenous alleles. The optimization-based correction step of HECIL is not biased by bases which have high frequency, and hence, is better able to capture variation between similar individuals. This is corroborated by the results obtained from testing HECIL on the highly heterozygous mosquito data set of *A. funestus*.

**Table 2:** Comparison of assembly-based metrics (with % improvement) evaluated from testing *E. coli*: with downsampled short reads (D-SR) having 18x coverage (lowest coverage) and original short reads, *E. coli* (Sequel-sequenced) *S. cerevisiae*, *A. funestus* (merged flowcells) on proovread, LoRDEC, CoLoRMap, and HECIL.

Data	Evaluation Metric	Original	proovread	LoRDEC	CoLoRMap	HECIL (Iter 1)	HECIL (Iter 5)
*E*. *coli* (D-SR)	# Contigs	182	29 (84.0)	28 (84.6)	24 (86.8)	**20 (89.0)**	—
	Largest contig	69,266	567,484 (719.2)	885,819 (1178.8)	813,262 (1074.1)	**1,204,631 (1639.1)**	—
	Total length	3,508,197	4,235,031 (20.7)	4,068,085 (15.9)	4,036,161 (15.0)	**4,596,013 (31.0)**	—
	N50	24,663	189,712 (669.2)	179,638 (628.3)	184,367 (647.5))	**232,826 (844.0)**	—
	NG50	17,847	212,621 (1091.3)	190,621 (968.0)	210,913 (1081.7)	**267,311 (1397.7)**	—
	Aligned base (%) - Ref/Query	83/84	87/89	92/93	48/92	**97/100**	—
	Average Identity (1–1) - Ref/Query	88/88	93/93	97/97	97/97	**99/99**	—
*E*. *coli*	# Contigs	182	26 (85.7)	24 (86.8)	**19 (89.5)**	**19 (89.5)**	**17 (90.6)**
	Largest contig	69,266	605,792 (774.5)	920,903 (1229.5)	1,089,140 (1472.4)	**1,223,474 (1666.3)**	**1,481,824 (2039.3)**
	Total length	3,508,197	4,629,719 (31.9)	4,623,137 (31.7)	4,624,793 (31.8)	**4,838,971 (37.9)**	**5,106,276 (45.5)**
	N50	24,663	231,774 (839.7)	226,456 (818.2)	239,066 (869.3)	**256,830 (941.3)**	**288,192 (1068.5)**
	NG50	17,847	231,774 (1198.6)	226,456 (1168.8)	239,066 (1239.5)	**294,635 (1550.8)**	**344,848 (1832.2)**
	Aligned base (%) - Ref/Query	82/87	92/92	98/98	54/94	**99/99**	**99/99**
	Average Identity (1–1) - Ref/Query	91/91	95/95	96/96	97/97	**98/98**	**99/99**
*E. coli* (Sequel)	# Contigs	84	34 (59.5)	29 (65.4)	29 (65.4)	**27 (67.8)**	**24 (71.4)**
	Largest contig	88,975	775,707 (771.8)	884,469 (894.0)	1,363,678 (1432.6)	**1,627,011 (1728.6)**	**1,865,932 (1997.1)**
	Total length	5,389,574	6,012,453 (11.5)	5,821,596 (8.0)	5,819,632 (7.9)	**6,374,798 (18.2)**	**6,773,369 (25.6)**
	N50	18,611	119,735 (543.3)	117,028 (528.8)	127,892 (587.1)	**141,213 (658.7)**	**162,580 (773.5)**
	NG50	13,903	116,255 (736.1)	113,036 (713.0)	118,087 (749.3)	**122,389 (780.3)**	**149,637 (976.2)**
	Aligned base (%) - Ref/Query	78/80	89/89	95/95	67/92	**97/97**	**98/98**
	Average Identity (1–1) - Ref/Query	88/88	92/92	92/92	93/93	**95/96**	**98/98**
*S. cerevisiae*	# Contigs	26	32 (−23.0)	28 (−7.6)	**24 (7.6)**	**24 (7.6)**	**23 (11.5)**
	Largest contig	1,543,990	1,537,979 (−0.3)	1,552,711 (0.5)	1,555,857 (0.7)	**1,558,190 (0.9)**	**1,713,201 (10.9)**
	Total length	12,341,981 (1.1)	12,485,995 (1.1)	**12,497,078 (1.2)**	12,315,869 (−0.2)	12,435,702 (0.7)	**12,731,203 (3.1)**
	N50	777,602	777,713 (0.0)	818,962 (5.3)	932,935 (19.9)	**1,018,591 (30.9)**	**1,308,313 (68.2)**
	NG50	777,602	777,713 (0.0)	818,962 (5.3)	932,935 (19.9)	**1,538,190 (97.8)**	**2,005,346 (157.8)**
	Aligned base (%) - Ref/Query	95/90	91/91	95/95	78/97	**99/99**	**99/99**
	Average Identity (1–1) - Ref/Query	92/92	93/93	97/97	98/98	**99/99**	**99/99**
*A. funestus*	# Contigs	998	712 (28.6)	788 (21.0)	847 (15.1)	**633 (36.5)**	**543 (45.5)**
	Largest contig	71,070	36,306 (−48.9)	75,298 (5.9)	72,306 (1.7)	**84,490 (18.8)**	**94,937 (33.5)**
	Total length	25,405,949	8,371,287 (−67.0)	26,745,092 (5.2)	26,802,126 (5.5)	**28,954,268 (13.9)**	**32,371,298 (27.4)**
	N50	13,038	14,802 (13.5)	15,118 (15.9)	14,555 (11.6)	**16,409 (25.8)**	**19,014 (45.8)**
	NG50	71,070	45,637 (−35.7)	77,294 (8.7)	76,306 (7.3)	**84,490 (18.8)**	**91,303 (28.4)**
	Aligned base (%) - Ref/Query	20/87	23/93	27/96	20/95	**31/99**	**37/99**
	Average Identity (1–1) - Ref/Query	83/83	87/87	95/95	92/92	**98/98**	**99/99**

For the case of HECIL, metrics are reported before and after using the iterative learning algorithm; specifically, iteration 1 (the core algorithm) and iteration 5 (with four rounds of learning) are shown.

Although the performance of hybrid correction algorithms largely depend on the set of high coverage short reads, we devise additional experiments to verify that restraining the coverage of short reads does not have a deleterious effect on HECIL. We down-sample short reads by randomly selecting 50%, 25%, and 12% of the data to be used for correction. In *E. coli*, this results in a subset of short reads for correction with an average coverage of 62×, 33×, and 18×, respectively. In Table 3, we present *k*-mer-based and alignment-based parameters from correcting long reads of *E. coli* with the down-sampled short reads using HECIL and in Table 2 we present assembly-based parameters from the lowest coverage (18x) of short reads. Thus, HECIL shows potential for use in projects that do not have high coverage short read data readily available: this is especially important in larger eukaryotic genomes sequenced predominantly with longer read technology.

**Table 3:** Comparison of *k*-mer-based and alignment-based metrics with downsampled *E. coli* short reads using HECIL’s core algorithm.

Evaluation Metric	All SRs	50% SRs	25% SRs	12% SRs
#unique *k*-mers	78,693,704	78,292,463	78,097,941	78,008,319
#valid *k*-mers	15,973,826	15,889,155	15,737,641	15,576,317
#aligned reads	31,332	31,328	31,322	31,318
#aligned bases	87,582,014	87,359,227	87,288,475	87,196,236
% matched bases	88.4	88.4	88.3	88.3
PI	99.7	99.7	99.7	99.6

HECIL can also be used to improve the results of alternative correction algorithms. To test its effectiveness, we assemble PacBio-sequenced long reads of E. coli with Canu and then use HECIL to further improve the quality of Canu-corrected reads for a new assembly. The results presented in Supplementary Tables S1 ans S2 show that HECIL consistently improved the assembled genome with respect to all the evaluation metrics.

In Table 4, we compare the runtimes and maximum memory usage incurred in correcting each data set (see Methods). proovread, LoRDEC, and CoLoRMap were run with 16 threads. The workload of HECIL was split into 16 concurrent tasks, which were run in parallel. Computation time of hybrid error correction methods is mainly dominated by the underlying steps of generating intermediate data, such as mapping short reads to the long reads. Similarly, LoRDEC and CoLoRMap construct a graph data structure, which demands high computational resources. LoRDEC, however, uses the efficient GATB library^26^, which lowers the overhead (see Table 4). Although our tool incurs higher computation time than LoRDEC, it is consistently faster (generally almost twice as fast) than the other correction methods and generates overall higher quality corrected long reads without a significant increase in memory consumption.

**Table 4:** Comparison of runtime and maximum memory footprint for correcting long reads.

Data	Method	Runtime (hh:mm:ss)	Memory (GB)
*E. coli*	proovread	6:15:37	11.4
	LoRDEC	38:53	6.2
	CoLoRMap	2:48:23	28.9
	HECIL (Iter 1; Iter 5)	1:16:55; 4:47:52	9.1; 9.1
4*E. coli* (Sequel)	proovread	42:53:06	34.6
	LoRDEC	17:47:27	24.3
	CoLoRMap	26:20:23	40.9
	HECIL (Iter 1; Iter 5)	19:33:47; 59:18:23	26.5; 26.5
4*S. cerevisiae*	proovread	20:54:15	14.5
	LoRDEC	3:43:12	6.1
	CoLoRMap	7:57:49	38.2
	HECIL (Iter 1; Iter 5)	5:14:09; 21:19:24	11.2; 11.2
*A. funestus* (Flowcell # 1)	proovread	76:13:47	8.8
	LoRDEC	35:08:13	3.1
	CoLoRMap	90:50:12	23.4
	HECIL (Iter 1; Iter 5)	46:06:47; 162:21:37	8.3; 8.3
*A. funestus* (Flowcell # 4)	proovread	36:32:25	7.3
	LoRDEC	11:25:05	6.7
	CoLoRMap	32:18:30	20.7
	HECIL (Iter 1; Iter 5)	17:38:01; 51:37:34	6.9; 6.9

Runtime includes index construction, alignment of short and long reads, and error correction (after the first and fifth iterations). Only the best and worst *A. funestus* results are shown.

### Effect of Iterative Learning

**Figure 1:**
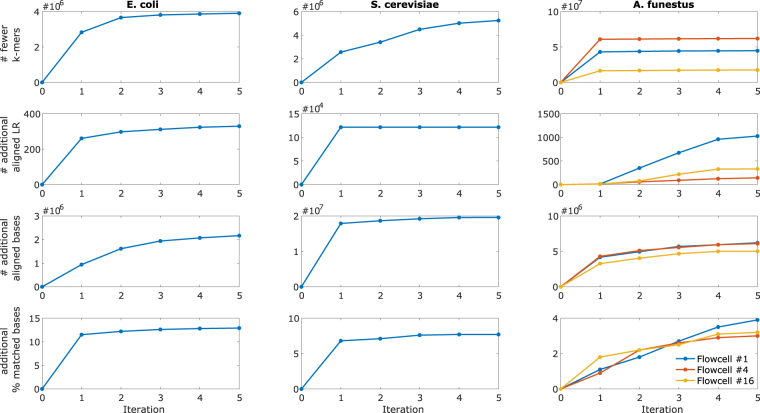
Improvement of alignment-based metrics (# fewer unique *k*-mers, additional aligned long reads, additional aligned bases, additional percent matched bases) for *E. coli*, *S. cerevisiae*, and *A. funestus* with iterative learning. The 0th iteration denotes the original data set and the 1st iteration indicates corrected data set obtained from running HECIL’s core algorithm.

We leverage our proposed iterative learning scheme on HECIL’s core algorithm to demonstrate its effectiveness in further improving correction accuracy. As discussed in the Methods section, we select a high-confidence cut-off of α = 95 percentile. The alignment-based incremental improvements obtained after each iterative correction of HECIL is presented in Fig. 1. For each data set (each column), we observe that the incremental metrics: number of fewer *k*-mers, number of additional aligned long reads, number of additional aligned bases, and additional percent of matched bases, improve after each iteration, until one of the termination criteria is reached. For the termination criteria, we select *ε* as 0.02 for the metric of unique *k*-mers. Based on this, we report alignment-based and assembly-based metrics obtained up to the fifth iteration of HECIL in Tables 1 and 2, respectively. HECIL in conjunction with iterative learning consistently outperforms all the evaluation metrics. For a few metrics, such as number of contigs in *E. coli* and *S. cerevisiae* and total length in *S. cerevisiae*, the core algorithm of HECIL is comparable but does not outperform the alternatives, and the iterative version of HECIL consistently results in better performance. These results verify the potential of the iterative learning-based component of HECIL, particularly in heterozygous samples like the mosquito data set used in this study.

## Discussion

Third-generation sequencing techniques, particularly Single-Molecule Real-Time (SMRT) sequencing, is revolutionizing modern genomics. The usefulness of current long read data, however, is restricted due to high sequencing error rates. Hence, it is crucial to correct long reads prior to downstream applications like *de novo* genome assembly. In this paper, we develop a novel approach of hybrid error correction called HECIL, which corrects erroneous long reads based on optimal combinations of base quality and mapping identity of aligned short reads. As seen in Tables 1 and 2, HECIL performs significantly better for an overwhelming majority of evaluation metrics, even with limited amounts of short reads available for correction. We show for the first time that our formulation can also be used to correct residual errors in *de novo* assemblies and therefore can be used to further polish existing long read assemblies where short read data are available. Spatial mapping information has been used very sucessfully in other areas of bioinformatics, such as protein function prediction^27,28^.

We speculate that the iterative procedure will improve the performance metrics that we are concerned with (for example, the number of unique *k*-mers) until saturation. Due to our confidence-based correction, after each iteration, the quality of alignment between the long and short reads are expected to improve, causing the normalized weight and the high-confidence threshold to increase until a saturation point is reached, beyond which it is unexpected that a significant improvement of the evaluation metrics will be seen. Note that this is a conjecture, laying a rigorous mathematical framework for proving iterative improvement likely cannot be made without making (possibly unrealistic) assumptions on stochastic properties of the normalized weights; this remains an open problem.

To the best of our knowledge, this is the first time an iterative strategy for improving correction quality via confidence-informed realignment has been proposed. The confidence-based iterative procedure shows potential using the HECIL core algorithm, but could also be seamlessly integrated with other error correction algorithms that leverage short read alignments since it is data-driven and algorithm independent. The current version of HECIL allows decomposition of the workload into independent data-parallel tasks that can be executed simultaneously. A natural extension of the tool will be to implement multi-threading to achieve speedup on traditional machines.

## Methods

Similar to existing hybrid error correction methods, HECIL requires all reads to be derived from highly similar individuals. We begin by aligning the given set of short reads to the long reads. For each alignment, we compute normalized weights using base quality information and alignment identity of the underlying short reads. The short read that maximizes the sum of these normalized weights is used for correction. In this manner, we tend to select higher quality short reads that have a suitable degree of overlap with a long read. This forms the core algorithm of HECIL.

Next, we optionally define a subset of these corrections as *high confidence* and correct only these high-confidence errors. By introducing elitism to the correction procedure based on confidence, the updated long reads now exhibit slightly higher consensus (or similarity) with the short reads. Therefore, we expect to obtain slightly higher quality alignments for fixing lower confidence corrections in subsequent iterations: this is the intuition behind the iterative learning procedure. Herein, we discuss each of these steps in detail.

### HECIL’s Core Algorithm

#### Quick Correction

**Figure 2:**
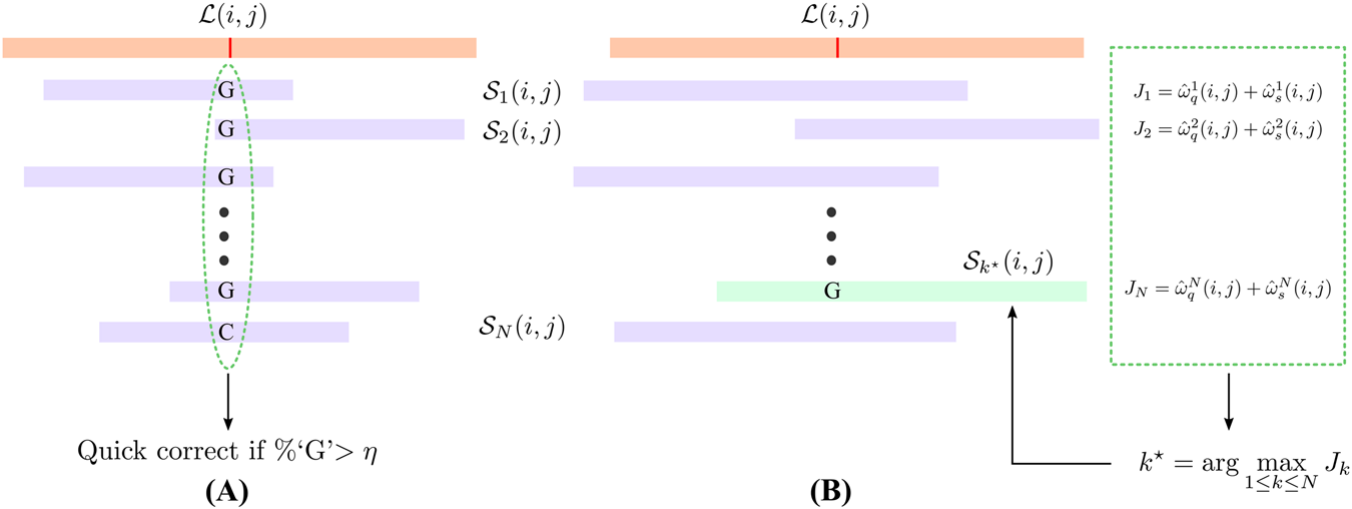
Illustration of HECIL’s core algorithm. The orange rectangle denotes an erroneous long read and the purple rectangles represent aligned short reads. (**A**) Quick correction with high consensus. (**B**) Optimization-based correction: The green dashed box depicts the objective function values, from which the optimal short read (green rectangle) is selected for correction.

We obtain read alignments using BWA-MEM^29^ with previously reported parameters^15,16^ and mark positions with disagreements (for example: mismatches, insertions, and deletions) on long reads as *questionable*. For each questionable position on the long read, we investigate the set of short reads that align to it. If there is strong consensus (determined by a threshold 0 ≪ η≤ 1 selected by the user), we replace the questionable base on the long read with the respective aligned base of the short read. This *quick correction* step is illustrated in Fig. 2(A). This step is inspired by majority voting methods^3^ and prior work^30^. Contrary to corrections based on a simple majority, we adopt a stricter threshold of at least 90% consensus (*η* = 0.9) to be eligible for quick correction. Shifting from majority voting to strong consensus prevents spurious corrections made on the basis of high-frequency, low-quality short reads. Note that quick correction also reduces the search space in the next step of HECIL’s core algorithm.

#### Optimization-based Correction

For the remaining questionable bases, we employ an optimization-based correction framework. Let ${\mathcal L} (i,j)$ be the *j*th questionable base corresponding to the *i* th long read. Suppose *N* short reads align to this ${\mathcal L} (i,j)$; ${\{{{\mathscr{S}}}_{k}(i,j)\}}_{k=1}^{N}$ denotes the set of aligning short reads. For each *k* = 1, 2, …, *N* we assign two normalized weights ${\hat{\omega }}_{q}^{k}(i,j)$ and ${\hat{\omega }}_{s}^{k}(i,j)$, representing the quality and similarity of the *k*th short read, respectively.

The normalized quality weight is given by

$${\hat{\omega }}_{q}^{k}(i,j)\,:\,=\frac{{\omega }_{q}^{k}(i,j)}{{{\rm{\max }}}_{1\le k\le N}{\omega }_{q}^{k}(i,j)},$$

where the scalar ${\omega }_{q}(i,j)$ is determined by extracting the PHRED quality score readily available from FASTQ files. The normalized similarity weight ${\hat{\omega }}_{s}^{k}(i,j)$ is obtained by calculating the alignment identity, defined as the number of exact matches of the *k*th short read ${{\mathscr{S}}}_{k}(i,j)$ to the long read ${\mathcal L} (i,j)$, divided by the length of ${{\mathscr{S}}}_{k}(i,j)$. Untrimmed short reads, therefore, may result in a lower estimated ${\hat{\omega }}_{s}^{k}(i,j)$, which is why we adhere to trimmed short reads in this study. For each short read, we compute a cost by taking a convex combination of the two normalized weights

1 $${J}_{k}(i,j)=\frac{1}{2}({\hat{\omega }}_{q}^{k}(i,j)+{\hat{\omega }}_{s}^{k}(i,j)).$$

We then solve the following optimization problem:

2 $${k}^{\ast }=\mathop{{\rm{argmax}}}\limits_{1\le k\le N}{J}_{k}(i,j).$$

which yields the index k^*^ of the short read ${S}_{{k}^{\ast }}(i,j)$ that exhibits the maximum combined quality and similarity weight. In case there is a conflict amongst maximizers, the short read with highest quality is selected to be the winner. Note that the optimal cost for each ${\mathcal L} (i,j)$ is denoted by ${J}_{{k}^{\ast }}(i,j)$. Subsequently, we replace the erroneous base ${\mathcal L} (i,j)$ on the long read with the corresponding base of the short read ${{\mathscr{S}}}_{{k}^{\ast }}(i,j)$. This procedure is illustrated in Fig. 2(B).

If perfect consensus (that is, η = 1 in Step 1) is reached amongst all the short reads, there is no need to perform Step 2, because both steps will yield identical corrections. Similarly, if we select a consensus threshold *η* ∈ (0, 1), then the probability that the quick correction value matches the optimization-based correction value is *η*, irrespective of the cost function selected. Therefore, choosing *η* close to 1 ensures that quick correction matches optimization-based correction with high-probability. We do not set *η* strictly equal to 1 hypothesizing that achievement of perfect consensus is rare in practice. Also note that the quality of a short read and its alignment identity with the long read are not contending objectives. That is, a high quality read does not always imply low similarity and vice versa. Therefore, we consider a convex combination of these objectives as in equation (2) rather than formulating a multi-objective optimization problem and searching for Pareto-optimal solutions.

### Improving Correction Performance via Iterative Learning

**Figure 3:**
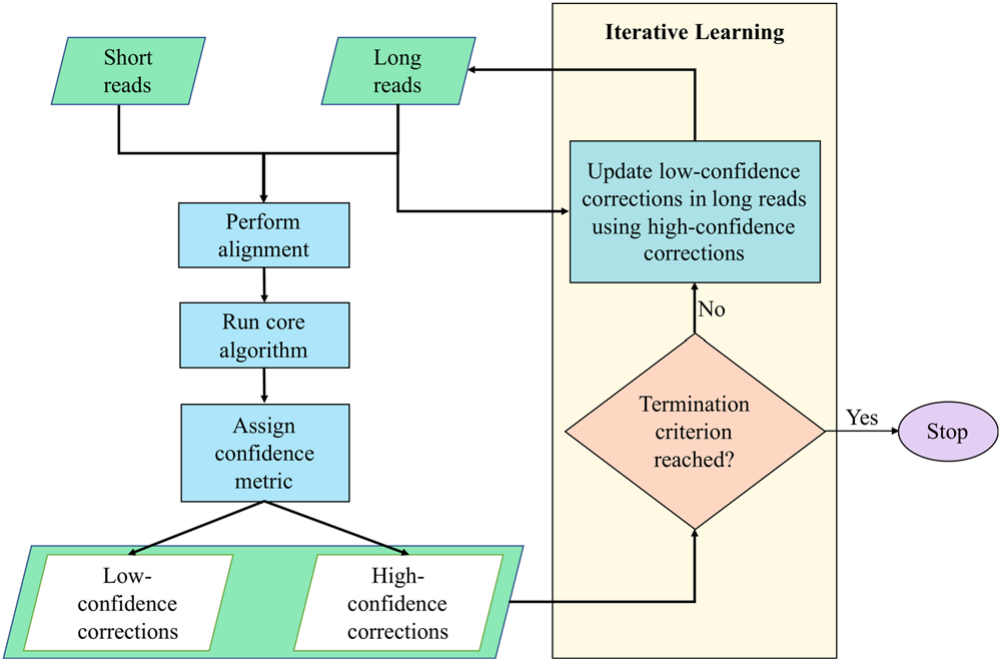
Iterative learning procedure of HECIL. Other hybrid error correction algorithms can replace the core algorithm.

A definition of iterative learning that closely resembles our proposed approach in this paper is offered^31^: iterative learning “considers systems that repetitively perform the same task with a view to sequentially improve accuracy”. Here, the *same task* refers to the core algorithm of HECIL, and the goal is to improve error corrections in the $\ell$th iteration by learning from high-confidence corrections in the $(\ell -1)$th iteration (see Fig. 3). An iterative approach has been previously used by iCORN^32^, which adopts a greedy method of correcting reference sequence and reverting them if mapping coverage reduces in successive iterations. A potential issue with iCORN is that corrections made in prior iterations can be reverted in subsequent iterations after realignment. HECIL eliminates this issue by selecting data-driven high confidence corrections that remain fixed in all subsequent iterations.

#### Assignment of confidence

For each ${\mathcal L} (i,j)$ in the $\ell$th iteration, suppose the corresponding optimal cost obtained by solving equation (2) be denoted by ${J}_{{k}^{\ast }}^{(\ell )}(i,j)$, and let ${\mu }^{(\ell )}$ denote the *α*-percentile (expressed as a scalar between 0 and 100) computed over all these optimal costs. Here we select *α* > 95 so that a small percentage of the optimal corrections are considered to be of high confidence. Selecting a high value of α ensures that only the highest quality corrections will always inform future iterations. Conversely, selecting α too close to 100 will result in slower improvement of correction accuracy, because large *α* implies that very few corrections are deemed high confidence. Therefore, the increment in information used to update the correction policy in the following iteration will be limited.

#### Realignment based on high-confidence corrections

We *learn* in successive iterations by realigning the updated long reads to the short reads. Note that, for each iteration, the updated context of ${\mathcal L} (i,j)$ could generate entirely different sets of aligned short reads, as well as disparate localized information from previous iterations, leading to the calculation of different sets of normalized weights ${\omega }_{{q}_{ij}}^{k}$ and ${\omega }_{{q}_{ij}}^{k}$. This is why the confidence threshold ${\mu }^{(\ell )}$ is recomputed based on the statistics of the optimal costs (namely, the percentile measure) and not fixed. The sites on the long read corresponding to low-confidence short reads are left to be changed via the core algorithm in a subsequent iteration while the high confidence changes in prior iterations are effectively fixed.

#### Termination criteria

We present the following termination criteria for the iterative learning procedure of HECIL. If the relative improvement in terms of unique *k*-mers between two successive iterations is below a given threshold *ε* ∈ (0, 1), that is,

3 $$\frac{\#{\rm{unique}}\,{k}-\mathrm{mers}(\ell -1)-\#{\rm{unique}}\,{k}-\mathrm{mers}(\ell )}{\#{\rm{unique}}\,{k}-\mathrm{mers}(\ell -1)} < \varepsilon ,$$

then we terminate after the $\ell$th iteration. Specific arguments why *k*-mers are used for termination are provided in the Results section in the context of *k*-mer-based evaluation metrics. To prevent a large number of iterations from occuring if *ε* is chosen to be very small in equation (3), we also recommend selecting a secondary termination criterion: the maximal number *n* of allowable iterations for iterative learning.

### Accession codes

https://github.com/NDBL/HECIL

## Electronic supplementary material

Supplementary Information
