# Supplementary material for: HECIL: A Hybrid Error Correction Algorithm for Long Reads with Iterative Learning
Source: Sci Rep. 2018 Jul 2;8:9936. doi: 10.1038/s41598-018-28364-3 (PMC6028576; doi:10.1038/s41598-018-28364-3)
Supplement: Supplementary file 1 — Supplementary Information [file 41598_2018_28364_MOESM1_ESM.pdf]

HECIL: A Hybrid Error Correction Algorithm for Long Reads with Iterative Learning

Supplementary Material

Olivia Choudhury*, Ankush Chakrabarty, Scott J. Emrich

Supplementary Figure S1:

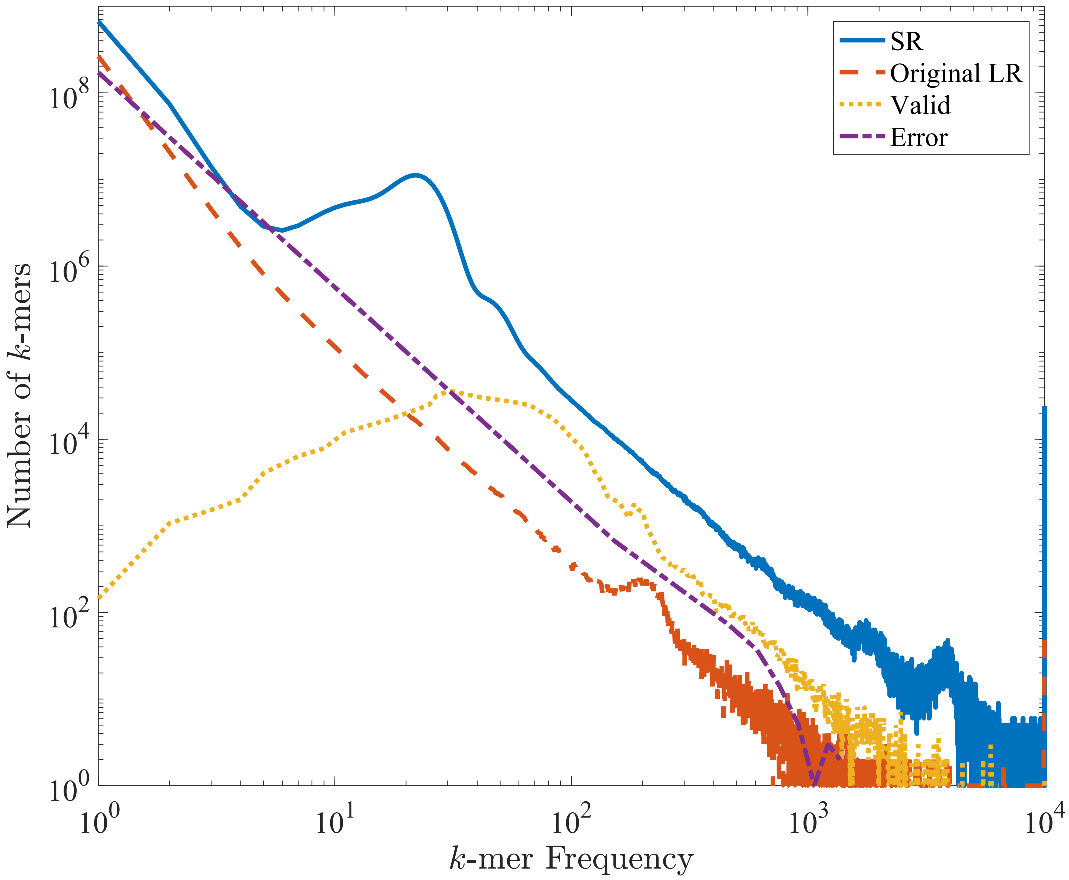

Figure S1: Distribution of k-mer frequency (k=17) in *Anopheles funestus* flowcell #16. The x and y-axes denote k-mer frequency and count of frequency, respectively. The blue line and dashed red line represent k-mers generated from short reads (SR) and original long reads (Original LR), respectively. The dotted yellow line indicates that majority of the valid k-mers have high frequency. The purple dot-dashes, representing error k-mers (not found in short reads), mostly consists of unique k-mers.

*Corresponding author: Olivia Choudhury: (ochoudhu@nd.edu)

Supplementary Table S1:

Data	Evaluation Metric	Original	Canu – Assemble	HECIL – Improve
E. coli	# unique *k*-mers	81,523,648	80,502,399	**78,849,104**
	# valid *k*-mers	14,531,881	8,407,389	**9,256,011**
	# aligned reads	31,071	29,862	**31,974**
	# aligned bases	86,642,500	84,395,516	**86,014,915**
	% matched bases	76.9	82.6	**84.8**
	PI	94.8	92.9	**95.7**

Table S1: Comparison of *k*-mer-based and alignment-based metrics evaluated after correcting long reads of *E. coli* with Canu and further improving with HECIL.

Supplementary Table S2:

Data	Evaluation Metric	Original	Canu – Assemble	HECIL – Improve
E. coli	# Contigs	182	38	**27**
	Largest contig	69,266	343,516	**559,641**
	Total length	3,508,197	4,585,942	**4,702,681**
	N50	24,663	176,245	**213,973**
	NG50	17,847	343,516	**356,014**
	Aligned base (%) - Ref / Query	83 / 84	89 / 92	**93 / 94**
	Average Identity (1-1) - Ref / Query	88 / 88	91 / 93	**94 / 95**

Table S2: Comparison of assembly-based metrics evaluated after correcting long reads of *E. coli* with Canu and further improving with HECIL.
